# Providing routine digital recordings of clinic visits to patients: a multiple-case study of three settings in the U.S

**DOI:** 10.1093/jamiaopen/ooag033

**Published:** 2026-04-03

**Authors:** Paul J Barr, Michelle D Dannenberg, Craig H Ganoe, Elizabeth Carpenter-Song, Reed WR Bratches, Meredith C Masel, Renata W Yen, Kerri L Cavanaugh, William Haslett, Rebecca Faill, Roger Arend, Sheri Piper, James Ryan, Glyn Elwyn

**Affiliations:** The Dartmouth Institute for Health Policy & Clinical Practice, Geisel School of Medicine at Dartmouth, Hanover, NH 03756, United States; The Center for Technology and Behavioral Health, Geisel School of Medicine at Dartmouth, Lebanon, NH 03766, United States; Department of Biomedical Data Science, Dartmouth College, Hanover, NH 03756, United States; The Dartmouth Institute for Health Policy & Clinical Practice, Geisel School of Medicine at Dartmouth, Hanover, NH 03756, United States; The Center for Technology and Behavioral Health, Geisel School of Medicine at Dartmouth, Lebanon, NH 03766, United States; The Dartmouth Institute for Health Policy & Clinical Practice, Geisel School of Medicine at Dartmouth, Hanover, NH 03756, United States; Department of Anthropology, Dartmouth College, Hanover, NH 03755, United States; Division of Nursing, The University of Alabama, Birmingham, AL 35294, United States; Oliver Center for Patient Safety and Quality Healthcare, The University of Texas Medical Branch, Galveston, TX 77555, United States; Department of Population Health & Health Disparities, UTMB School of Public & Population Health, Galveston, TX 77555, United States; The Dartmouth Institute for Health Policy & Clinical Practice, Geisel School of Medicine at Dartmouth, Hanover, NH 03756, United States; The Center for Technology and Behavioral Health, Geisel School of Medicine at Dartmouth, Lebanon, NH 03766, United States; Vanderbilt Center for Effective Health Communication, Vanderbilt University Medical Center, Nashville, TN 37232, United States; Department of Medicine, Division of Nephrology, Vanderbilt University Medical Center, Nashville, TN 37232, United States; The Center for Technology and Behavioral Health, Geisel School of Medicine at Dartmouth, Lebanon, NH 03766, United States; Department of Biomedical Data Science, Dartmouth College, Hanover, NH 03756, United States; Patient Partner, United States; Patient Partner, United States; Ryan Family Practice, Ludington, MI 49431, United States; The Dartmouth Institute for Health Policy & Clinical Practice, Geisel School of Medicine at Dartmouth, Hanover, NH 03756, United States

**Keywords:** Clinic visit recordings, patient-centered communication, Implementation (barriers and facilitators), Caregivers, Qualitative Research

## Abstract

**Objective:**

To explore the impact, barriers, and facilitators of routinely sharing clinic visit recordings with patients in diverse clinical settings.

**Materials and Methods:**

We conducted a multiple-case study of three early-adopter clinics in the U.S.: a primary care clinic in Michigan and an oncology clinic in Texas that shared audio recordings, and a neurology clinic in Arizona that shared video recordings. From March 2016 to January 2017, we conducted semi-structured interviews with clinicians, patients, care partners, and administrators (≥18 years, English-speaking), and direct observation of patients using their recordings. Transcripts were analyzed using framework analysis to identify cross-cutting themes. Three coders independently reviewed all transcripts, and a medical anthropologist audited key analytic stages.

**Results:**

We interviewed 67 stakeholders (32 patients, 10 care partners, 15 clinicians, and 10 administrators). Across sites, stakeholders reported that recordings improved patients’ recall, understanding, and communication. Patients also used recordings for reflection on their performance in visits and planning, while care partners described reduced anxiety and enhanced involvement. Clinicians reported improved visit interactions, and some used recordings for self-assessment. Key factors influencing implementation included clinic culture, institutional support, workflow logistics, data security, and patient characteristics. Concerns were limited and focused primarily on data privacy. A conceptual framework summarizing themes related to barriers, facilitators, use, and impact of routine recording in healthcare was developed.

**Discussion:**

Routinely sharing visit recordings can enhance patient-centered communication and care partner engagement while supporting clinician performance. Successful implementation depends on aligning institutional culture, privacy safeguards, and workflow integration.

**Conclusion:**

Sharing visit recordings was acceptable and beneficial across stakeholders. The practice of sharing recordings revealed that clinic visit interventions are more than just transactions of medical information—they promote emotional support, self-reflection, and family engagement.

## Background and significance

With smartphones enabling easy and high-quality recording, patients and clinicians are increasingly recording visits for personal use, and some clinics now offer routine visit recordings.^1^ This trend is expected to grow with the widespread adoption of ambient visit recording, which utilizes advances in natural language processing (NLP) to support automated generation of electronic health record notes and clinical decision-making.^2^ Access to visit recordings has been shown to improve recall, understanding, and patient satisfaction.^3^^,^^4^ In prior studies, 71% of patients listened to their recordings, and 68% shared them with a care partner.^4^ Recordings may provide benefits beyond being a durable resource of visit information: reducing the number of phone calls made by patients to the clinic,^5^ increasing patient confidence in their treatment,^3^ and offering emotional support.^6^^,^^7^ Recording use also appears to increase information sharing with family members and fostering greater family engagement.^6–10^

While patients generally support visit recordings, the views of clinicians and health systems are mixed. A 2017 survey found 28% (129/456) of U.S. clinicians reported recording a clinic visit for a patient’s personal use.^1^ In a more recent survey of 360 oncologists, 93% reported experiencing a patient recording, 96% allowed recordings, 75% were comfortable with this practice, and 77% believed it improved the patient experience.^11^ However, 50% raised concerns that recordings can negatively influence the disclosure of sensitive information.^11^ Other clinicians raise medico-legal concerns related to recordings and potential disruption to the patient-clinician interaction.^1^

Despite growing interest in visit recording, prior studies have mostly been controlled clinical trials, with none evaluating its use in everyday clinics.^4^ The emergence of clinics routinely offering recordings has enabled our team to conduct the first study of real-world adoption outside research settings.

## Objective

We report on a multiple-case study of clinics routinely sharing recordings with patients, where we explored how recordings were integrated into clinical workflows and examined implementation barriers, facilitators, and stakeholder experiences.

## Materials and methods

### Design

We used a multiple-case design, conducting semi-structured interviews (March 2016–January 2017) at three clinics that routinely share visit recordings. All participants gave informed consent. Institutional Review Board (IRB) approval for the research was obtained from the Committee for the Protection of Human Subjects (CPHS) at Dartmouth College (STUDY#29380).

### Settings

An internet search in 2014 identified three clinics routinely offering patients access to recordings (see Table 1). Within these health systems, recording use varied by clinic and clinician. Research team members (PJB, MD) conducted five-day visits at each site. Descriptions of clinic settings and recording procedures reflect the time of the case study; platform screenshots (used at two sites) are included in the Supplementary Material.

**Table 1.** Visit recording logistics at each site.

Patient population	Recording access	Recording stored by the clinic	Recording initiation	Additional notes
Single practice, primary care. MI	Audio recorded using a computer. Patient access online through a secure account	Yes	Clinic initiated	The clinician invited patients to participate in a recording program and documented their written consent (see Supplementary Material). Several patients reported that they consented to access the electronic system but needed to be made aware that recordings were available. During visits, clinicians annotated recordings in real time by entering notes into the EHR, which were timestamped, that is, 07:52:00 Added problem “Backache (finding)”
Oncology Dept. TX	Audio recorded using a digital recording device. Patient access on the device.	No	Patient initiated	At their first visit, patients received a digital recorder with verbal and written instructions. While typically used during the initial visit, both patients and clinicians reported infrequent use afterward, especially for procedural visits like chemotherapy administration. Half of clinicians said they had recorded visits prior to the program’s launch (see Supplementary Material)
Neurology Dept. AZ	Video recorded using an electronic tablet. Patient access online. Patient access online through a secure account	Yes	Clinic initiated	Clinicians or medical assistants introduced video recording, and interested patients created an account and gave consent (see Supplementary Material). Some clinicians recorded the entire visit, whereas therapists reported starting and stopping the recording to capture specific exercises, for example, sit-to-stand exercises. Patients could share videos only with care partners who created their own accounts.

#### Site 1: Primary care practice, Michigan, USA

A rural single-clinician practice offering audio recordings since 2014 (19 months). The clinician developed a HIPAA-compliant system with indexed playback linked to visit notes (eg, medication changes) and patient-centered tools (eg, goals of care, pain scale).

#### Site 2: Oncology practice, Texas, USA

Since 2009 (5 years), new patients at the oncology and breast health clinics of an academic center have received digital recorders, guidance on their use, and encouragement to bring them to each clinic visit. Recording was not standard elsewhere in the health system.

#### Site 3: Neurology/neurosurgery practice, Arizona, USA

Video recordings have been provided since 2008 (6 years) via a commercial platform developed by a clinic neurosurgeon in this academic tertiary medical center. Stored on a HIPAA-compliant cloud server, recordings can be shared with care partners and other clinicians. Initially offered in neurosurgery, the service expanded to other departments (eg, Parkinson’s rehabilitation). Recording was not standard elsewhere in the health system.

### Participants

Three qualitative researchers (PJB [male, PhD, project lead], MD [female, MPH], and CG [male, user-centered design expert]) conducted interviews with four stakeholder groups: (1) patients, (2) care partners (family/friends with access to recordings), (3) clinicians, and (4) clinic administrators. Eligible participants were ≥18 years and English-speaking. At least six interviews per stakeholder group were conducted, with representation coming from each site.^12^ Site champions distributed study information; interested patients were contacted by the study team for consent and provided contact details for care partners. Clinic staff were recruited via email. All participants received a $20 honorarium.

### Data collection

Following the guidance of Yin,^13^ our principal evidence source for information on visit recording was semi-structured interviews, supplemented with direct observations of patients using their recording systems:

*Semi-structured interviews*: Semi-structured interviews were conducted by researchers trained in qualitative methods (PJB, MDD, CG). Interviews were mostly during site visits in a private clinic room; care partner interviews were often conducted via telephone. Interviews were 30 minutes on average and were audio-recorded for transcription and analysis. Interviews consisted of the following main sections: recording implementation (eg, motivation to record, logistics), recording use (eg, by patients, clinicians, care partners), benefits or concerns about visit recording, and the recording platform (eg, ease of use).

*Direct observation:* At primary care and neurology sites, patients willing to demonstrate the online platform did so before their interview. Using concurrent think-aloud techniques, ^14^ they accessed recordings while describing each step. Screen activity, input, and audio were captured with TechSmith Morae software.

### Data analysis

Interviews were transcribed verbatim by Acusis^®^.^15^ Data collection and analysis occurred concurrently until thematic saturation.^12^ Using ATLAS.ti, we applied a five-step framework approach: familiarization, framework development, indexing, charting, and interpretation.^16^^,^^17^ Major themes reflected common ideas across sites; minor themes captured less frequent but relevant concepts. Three researchers (PJB, MDD, CG) developed and refined a codebook, with disagreements resolved by GE. Emergent codes were incorporated iteratively. A medical anthropologist (ECS) reviewed all steps to ensure analytic rigor.

## Results

### Participant characteristics

We conducted 67 interviews with 32 patients (mean age 63 [SD 12]; 12 female), 10 care partners (mean age 57 [SD 13]; 9 female), 15 clinicians (mean age 48 [SD 11]; 7 female), and 10 clinic administrators (mean age 43 [SD 13]; 9 female) (Table 2). Two oncology patients and three neurology clinicians declined to record despite awareness.

**Table 2.** Interviewee demographics.

	PCP	Oncology	Neurology	Total
Stakeholder	*N* = 24	*N* = 16	*N* = 27	*N* = 67
Patient	16 (67%)	6 (38%)	10 (37%)	32 (48%)
Care partner	5 (21%)	2 (13%)	3 (11%)	10 (15%)
Wife	2	–	2	4
Husband	1	–	–	1
Adult Child	2	1	1	4
Parent	–	1	–	1
Clinician	1 (4%)	4 (25%)	10 (37%)	15 (22%)
Clinic Administrative Staff	2 (8%)	4 (25%)	4 (15%)	10 (15%)
Gender				
Female	12 (50%)	12 (75%)	14 (52%)	38 (57%)
Male	12 (50%)	4 (25%)	13 (48%)	29 (43%)
Mean age (SD) [Range] Overall	61 (13) [36-80]	49 (13) [30-73]	55 (15) [26-79]	56 (15) [26-80]
Education				
High School Grad or Less	5 (21%)	3 (19%)	1 (4%)	9 (13%)
Some College, associate, or bachelor’s Degree	14 (58%)	7 (50%)	13 (48%)	34 (51%)
Master’s, Doctorate, or Professional	5 (21%)	5 (31%)	13 (44%)	23 (34%)
Missing	—	1 (6%)	—	1 (2%)
Race				
White	23 (96%)	9 (56%)	22 (81%)	54 (81%)
Black or African American	—	3 (19%)	1 (4%)	4 (6%)
Asian	—	1 (6%)	—	1 (2%)
American Indian or Alaska Native	1 (4%)	—	—	1 (2%)
Other	—	2 (13%)	2 (7%)	4 (6%)
Missing	—	1 (6%)	2 (7%)	3 (4%)
Ethnicity				
Non-Hispanic	22 (92%)	11 (69%)	22 (81%)	54 (81%)
Hispanic	1 (4%)	3 (29%)	5 (19%)	10 (15%)
Missing	1 (4%)	2 (13%)	—	3 (4%)
Language Spoken				
English	23 (96%)	14 (88%)	25 (93%)	62 (93%)
English & Spanish	—	2 (13%)	2 (7%)	4 (6%)
English & Cherokee	1 (4%)	—	—	1 (2%)

We observed eight patients (seven in primary care, one in neurology) using recording systems. Among 32 interviewed patients, 30 had recorded at least one clinic visit. Of these, 28 listened back; one was unaware of the recording, and another had technical issues but shared it with a care partner. Most listened within days, and some before subsequent visits. Sixteen (52%) patients reported that a care partner listened, including seven (22%) who reported sharing with multiple care partners, and often they listened together. All interviewed care partners had listened. Patients in specialty care were more likely to review recordings multiple times compared to those in primary care.

### Barriers and facilitators to implementing recording in practice

#### Barriers

Logistical and technical challenges especially setting up the video recording, disrupted clinic flow and increased staff burden. In oncology, busy patient check-ins made it difficult for staff to introduce recordings, “*just the volume of the clinic. The MAs and the nurses get busy, and sometimes they do forget*” (Admin_01_oncology). Patients using digital recorders sometimes forgot to bring them back for future visits, *“I guess it’s kind of—I guess you really don’t think about bringing a tape recorder to your appointment*” (Admin_01_oncology). Yet these patients reported recording elsewhere, unlike the other sites, likely due to the transportability of the recorder. In Neurology and primary care, limited access or patient ability to use the Internet were barriers to recording use, “*I think it’s a technology thing. Yeah. Not, not a privacy thing*” (Clinician_10_neurology). User demonstrations revealed that online systems were user-friendly; however, long load times and a lack of a password reset option were issues with the recording platform in primary care.

*Clinic culture* posed a barrier as recording is not typical in US medical practice, “*novel phenomenon, it requires extra sensitivity to how it’s introduced*” (Clinician_01_pcp). Relatedly, one clinician mentioned the absence of Medicare reimbursement as a barrier. Data security, liability, and recording integrity were raised. Clinicians did not want to be responsible for recording data, were concerned that patients might edit the recordings, and the possibility of discrepancies between a recording and their written note. Finally, clinicians were concerned about the ability of some patients to access and use recordings, such as those with limited cognitive ability, but acknowledged that these patients may benefit the most from recordings.

#### Facilitators

The *ease of system use* was a significant facilitator. Despite logistical challenges, recording systems were considered user-friendly, and recordings were available within 24 hours. Patient demonstrations showed they could easily access and navigate their recordings, particularly in primary care where annotation features were noted as helpful. In both systems, patients could leave a note on their recordings.

A *clinical champion* and *institutional support*, such as recording set-up support, facilitated use. Trust between the doctor and patient also facilitated recording use: “*I know that he wouldn’t be involved in something unless he thought it was going to be to the benefit of the patients in his practice*” (Patient_15_pcp) and “*Trust. Well, we just—I guess one reason is because we completely trust Dr. X*” (Carepartner_13_pcp).

At the neurology site, recording was incentivized; clinicians who record receive a 10% reduction in malpractice premiums and improved maximum coverage: “*I have a policy for $3 million to $1 million, which means any particular case is $1 million and it is a maximum of $3 million in a year… So, three separate cases. But if I get sued above a million, then I’m on my own. So, now they are giving me $2 million of protection*” (Clinician_06_neurology). Recordings were also more likely where the perceived benefit based on *patient characteristics* was high and information recall could be challenging, for example, complex treatment regimen or cognitive impairment.

### Thematic analysis of stakeholders’ experience with routine visit recordings

From our qualitative analysis of interviewees’ experiences with routine visit recording, we identified five major and seven minor themes. Below, we elaborate on these themes using sample quotes in Tables 3 and 4.

**Table 3.** Major themes from interviews and sample quotes.

Major themes	Sample quotes
Broad support for recording from all stakeholders	“*I mean there’s no drawback… I thought it was the best idea that could ever be used, and I think it should be used in other situations*” (Patient_01_oncology). “*patients come to see their oncologist, the medical terminology, some of the providers don’t use layman’s terms, of course they don’t retain all of the information, so then going home and listening to that recording again, taking it home to their family members, it helps tremendously and we encourage them to bring them to all of their appointments*.” (Admin_01_oncology) “*It’s really beneficial because my mom probably would not be able to understand 50% of what he said to her at her appointments, where I can just login and read, “Wow what’s going on,”…*” (carepartner_19_pcp)
Recordings promote greater recall and understanding	“*Understand more about my cancer and you know the different medications that I’m taking because I could always go back on the computer and look up the medication and see what the side effects are and how I’m going to be feeling. So that’s what the recording—it helps a lot because sometimes if I didn’t have a recording, I couldn’t remember what he said*.” (Patient_02_onclology) “*but when they gave me the recorder, it took a lot of pressure off of me in the sense that I could play it back to understand what the doctor has said and the questions that I asked and then the questions that I needed to ask in order to relieve some pressure off of me, so I could understand what was going on*” (Patient_01_oncology) “*I think to go back and actually, you know, pay attention to what was said, sometimes a lot of information gets moved along very quickly and you can’t really remember what the doctor’s comment was on something, you go back and listen, there it is*.” (Patient_12_pcp)
Recording used to reflect on the patients’ contributions to the visit	“*I would go back and check it out, did I tell him everything I wanted to tell him?*” (Patient_11_pcp). “*And she (the patient) was often reporting things to her doctor that I (patient’s daughter) didn’t think were very accurate.*” (carepartner_16_pcp). “*Now, I can actually go back and hear it, but remembering what I said and if I didn’t say, exactly what I missed or if I wanted to elaborate on something, that would give me the opportunity to do that.*” (patient_09_pcp) “*I think, by a doctor listening to it can say “Well, I should have said this” or “Next time, I want to do this,” or the patient listens to it and say, “Oh, I didn’t tell him this part of what was wrong with me today*.” (patient_15_pcp) “*What I do is I write reviews of what he says…I can sit down and write what I think about what we discussed in our last appointmen*t.” (patient_9_pcp)
Sharing recordings with care partners facilitated engagement in patient care, especially for care partners who could not attend visits	“*It’s really beneficial because my mom probably would not be able to understand 50% of what he said to her at her appointments, where I can just login and read, “Wow what’s going on,” and he is very concerned about certain issues. I do bring some of those up to her*” (Carepartner_19_pcp) “*my other daughter, when we get together and we will turn it on and listen and then she would say, “Mother did you understand everything on the recording that Dr. Mallon had told you?””* (Patient_02_oncology). “*the patient might not be able to understand it for themselves or be afraid to even listen to it. Maybe don’t wanna deal with it, but a caregiver of course—I mean the caregiver can’t be calling the doctor every minute and getting this information, so the information is made available and they are able to, you know, vet if off very quickly and understand it*” (Patient_15_pcp). “*one of our sons was here from South Dakota and so we invited him to watch it and he is a PA so he was very interested, plus it’s his father*.” (Carepartner_02_neurology) “*the ones that I can’t come to [visits] are the ones that I watch*” (Carepartner_01_Neuro) “*Kind of like when you’re doing research and you need more information on the subject you’re talking about. It helps me better, and be more informed to help him out [the patient]*.” (Carepartner_17_pcp)
Prepare for future visit	“*that way you could look at later on for future meetings and if I want to know more about this or write a note you can be able to talk to your doctor about it or remind you what you need to say because normally, you only see the doctor for less than 15 to 30 minutes. So if you look at by the time you get to talk to him with 2 minutes that gives you 13 minutes to actually say what your problem is*” (Patient_01_neurology) “*It does help me to prepare for the next visit and my treatment as well at the same time. Because I can listen to it before my appointment and knowing if there is an issue that I have with the previous appointment*.” (Patient_07_pcp) “*you have the same conversations multiple times and/or a different caregiver comes or the spouse comes and then you spend the whole beginning of the next session talking about what you already talked about. This way, it’s kind of a time-saving opportunity during the one-on-one sessions plus it gives them the opportunity to review it in between sessions*.” (Admin_01_neurology)

**Table 4.** Minor themes from interviews and sample quotes.

Minor themes	Sample quotes
Recordings were perceived as valuable as the conditions became more complex	*When she is not having as much difficulty, I don’t feel the need to listen.*” (Carepartner_16_pcp)
Patients have the right to a recording	*I think that they should have a recorder and offer it, and I couldn’t care if the doctor liked it or not because I’m the patient”* (Patient_01_Oncology). “*I personally think it’s a good service. I think it’s good for the patient. I—I think it’s a patient’s property. So, what the patients do about their own information, it is their concern.*” (Clinician_02_oncology).
Video recordings superior to written notes	“*the people who have bought into it [video] do their exercises better and more consistently*” (Clinician_07_neurology). “*I think especially in our room when there is a physical activity, it’s really crucial for them to have the video… with Parkinson’s disease in particular, the right kind of exercise at the right intensity is what is most effective in minimizing the impact of the disease.* ” (Admin_01_neurology)
Recordings used a means of emotional support for patients and care partner	“*it’s like a right hand that I can go back and forth, and if he tells me good news, then I can play it back and forth when I’m down in the dumps*.” (Patient_01_oncology). “*just nice to hear… I can hear even my voice, you know what it is, if it’s depression or something. Wow, look at how I sounded there and then a year later, look at where I am at after the treatment and all that*” (Patient_08_pcp)
Improve clinic visit interaction	“*That gives me a sense of mental freedom to really be with that person more*.” (Clinician_01_pcp). “*I guess that might assure us that there isn’t any—that his care isn’t affected at all negatively or maybe positively because he knows it’s being recorded*” (Carepartner_13_pcp) “*I think it might improve it. I think because if the patient is feeling—is aware of this and feeling any way different that they should perform in how they speak to the doctor or how clear they are or whatever, I think at the same time the doctor is feeling the same need. To make sure that he or she is being clear to the patient*.” (Patient_15_pcp)
Improve clinician practice	“*I remember watching myself do a treatment session and I was giving way more of verbal cues than I thought necessary and I had no idea*”. (Clinician_03_neurology) “*So, in the middle of the surgery, and I looked at my note and my note didn’t say whether they had symptoms there or not. I pulled up the video and found, yeah the patient did have symptoms, but I didn’t have in my note*”. (Clinician_06_neurology)
Protective for clinicians	“*I also view it as a protective for me because patients frequently say you didn’t tell me this, and more often than not I did.*” (Clinician_06_neurology).

### Broad support for recording from all stakeholders

Stakeholders supported recording, some believing it should be a patient’s right, reporting benefits, and few expressed concerns.

### Recordings promote greater recall and understanding and were also used to prepare for future visits

Key benefits of sharing recordings included improved recall and understanding of visit information. Patients reported being better able to “absorb” and “clarify” information, follow instructions, and manage complex conditions by revisiting recordings on their own time. Video recordings, especially of speech and physical therapy, were viewed as more effective than written instructions. Several patients used recordings to prepare for future visits. One clinician also noted the opportunity for a visit recording to become an important “historical” artifact to be used by families in the future.

### Recording used to reflect on the patients’ contributions to the visit

Patients and their care partners commonly reported listening back to assess patient contributions to the visit, describing this as “*interacting with yourself*” (Patient_15_neurology). Relatedly, some participants used the recording as a form of emotional support, for example, hearing the improvement in their condition was reassuring.

### Sharing recordings with care partners facilitated engagement in patient care and reduced anxiety

Sharing recordings with care partners was viewed positively by all stakeholders, as it supported monitoring, tracking, and family engagement in care. Recordings helped care partners recognize signs of worsening health and provided access to detailed, accurate information from visits. This was especially helpful for care partners who could not attend visits (ie, working, living in a different location). Recordings reduced care partner anxiety by enabling access to information about the patient’s health condition and facilitating better conversations at home with the patient “*It reconfirms the accuracy of our understanding of what transpired during the visit and Linda—where Linda and I together form a full brain and we’re able to stay on top of our lives* (Patient_13_ pcp)”. Despite the benefits, almost half of the patients did not share recordings, mostly in primary care, citing a lack of care complexity. Most patients would share if their condition worsened or the complexity increased.

### Clinicians’ use of recordings was less common, but its potential as quality improvement tool and for legal protection emerged

Recording use by clinicians was mixed. Five clinicians in primary care and neurology used recordings, while none did in oncology, where patients retain recordings on digital recorders and clinics did not retain a copy. Clinicians used recordings to assist with documentation, billing, and to reflect on their clinical practice. Two clinicians mentioned reviewing recordings during surgical procedures to clarify information not adequately captured by notes. Two clinicians mentioned sharing recordings with colleagues to assist with patient care. Clinicians also reported that recording reduced follow-up calls and enhanced legal protection by having an accurate record of the clinic interaction.

### Presence of recording had no negative impact on the visit and awareness that recordings would be shared likely improved communication

Most interviewees did not feel that recordings negatively affected their visit; some felt recordings improved their visit interaction as they could be more present and review the recording later. Three clinicians in the study chose not to record, citing concerns about intrusiveness; however, these concerns were not realized by clinicians who did record. Clinicians reported that recordings appeared to enhance communication by reducing information overload and heightening the clinician’s sense of accountability. There was also a sense that recordings eventually faded into the background “*I think it sort of goes out of our minds, the thing is in the background and then we just keep talking. I think at the—I’m looking back at my own feeling, I think I just try to be a little bit more formal, try to talk with the persons who might be listening, but that sort of goes away and then I think it really does not make a whole lot of change towards them*.” (Clinician_02_oncology)

### Concerns were not commonly reported

Despite probing for privacy, confidentiality, and security issues, most interviewees raised no concerns. Two patients, however, expressed minor concerns, including not wanting to receive “*solicitations*” (Patient_13_pcp), based on the recorded information. One clinician raised a concern about the unknown consequences of applying Artificial Intelligence to recordings “*It is one of the things that I’m thinking about just last night is what are we really unleashing, and we don’t really know what this deep learning or this new machine learning things really are. I mean they seem like cool to me as all sophisticated ignorant type of a course that just knows enough to get us say the words, but not really know enough to understand the full potency behind them*” (Clinician_01_pcp).

### Recordings of the future—highlighting essential visit information would be highly valued

All interviewees supported the possibility of technology automatically identifying key parts of the visit, as it would allow easy navigation of recordings. It was also noted that clinicians could use this feature to quickly and efficiently find information for notetaking or to prepare for future visits. Concerns were raised were related to mis-tagging leading to missed key information, too many tags, and patients over-focusing on just the highlighted information “*Well, I guess if the tagging was incorrect. Yes, that would be a concern. That would—I don’t want to say bad words, that would piss me off*” (Carepartner_02_Neurology). The information considered most important to highlight included medications, treatment plan (eg, follow-up, referral, lab work), diagnosis, and test and imaging results.

## Discussion

**Figure 1.**
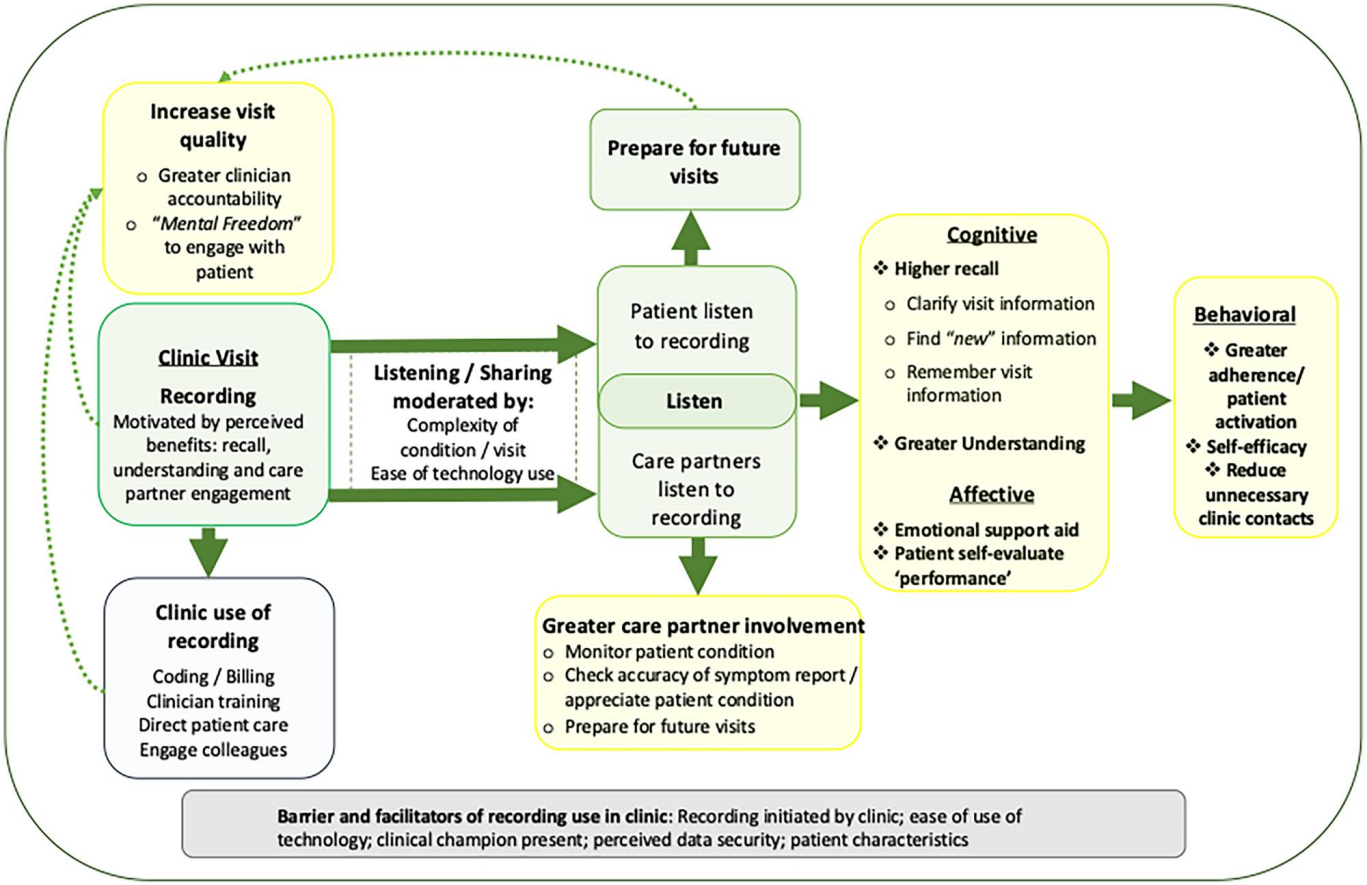
Conceptual representation of themes related to the use and impact of visit recording.

This is the first study to observe the routine sharing of visit recordings outside of research settings. Findings offer novel insights into the experiences of clinicians and care partners, with stakeholders largely viewing the recordings positively. Recording use was linked to disease severity and mode of providing recordings (internet-based resulted in higher use). Recordings offered more than a transaction of medical information, supporting quality improvement, emotional support, disease monitoring, and self-reflection (see Figure 1).

### Comparison to prior literature

Our findings, that patients perceived recording as improving recall and understanding, support those in controlled research settings.^3^^,^^4^ The advantages of video for physical therapy in neurology clinics support prior research.^18^^,^^19^ Patients mostly listened to recordings within the day after their visit; however, listening and sharing in primary care were less common than in specialty clinics. This was due to internet access issues in this rural. community, and a lack of disease complexity. Liddell and colleagues also found that a large proportion of primary care patients did not listen to recordings (39%), citing a lack of complexity.^20^

Beyond information recall, a novel finding was that patients used recordings to reflect on their performance and prepare for future visits. This suggests that patients are aware of their role in and are interested in ‘performing’ well during the visit. Patients mainly focused on what they forgot to say, as it may be important for their care. While audit and feedback are common for clinicians, similar strategies for patients are rare.^21^ Providing an opportunity for patients to reflect on their performance may be a low-barrier intervention to improve visit interactions.

Recordings promoted care partner engagement and facilitated patient–care-partner conversations. Similar findings were found when patients and care partners had access to visit notes in the patient portal.^22^ Patients mostly listened with their care partner when sharing recordings rather than in isolation. Recordings also provided emotional support for patients, replaying positive news from the visit, and provided care partners with a sense of reassurance. These findings support the work that hypothesizes the possible therapeutic benefit of recordings.^23^^,^^24^ Ryan et al.^6^ recently found that self-initiated recordings by in-patients served as reflection tools, promoting psychosocial support and connecting patients with their social networks. Recent studies^25^^,^^26^ have shown that recordings may enhance psychological well-being and reduce anxiety—like effects seen with care partner access to patient portals.^22^^,^^27^

Our findings align with prior studies, which show clinician support for recordings despite initial hesitation.^3^^,^^28^^,^^29^ In our study of clinicians who routinely record, they noted improved communication, citing a heightened sense of accountability. This supports earlier work, which reported increased attentiveness and thoroughness of clinicians during recorded visits.^3^^,^^6^ Both patients and clinicians described reduced cognitive burden and less pressure to take notes in the moment, as a recording would be available. For clinicians, recordings provided “*mental freedom*” to be completely present, as they could be used for generating notes later.

Using recordings for clinician self-reflection and to engage other colleagues in improving care delivery is novel but aligns with practices in clinical training, such as those in psychiatry, where recordings are often used to review performance.^30^ Weiner et al.^29^ have demonstrated the high feasibility of providing feedback to clinicians using recordings that can enhance patient-centered communication.

Finally, from a systems perspective, reduced malpractice premiums and increased coverage for recording clinicians align with legal views in the early 2000s that recordings would be protective of clinicians.^31^ Our findings also support prior work showing that recordings reduce follow-up calls, as many patient queries relate to clarifying visit details.^5^^,^^10^ Additionally, some patients and clinicians, unprompted, expressed the belief that patients have a right to their visit recordings, echoing recent survey and qualitative research findings in the U.S. ^11^ and Australia.^6^

### Strengths and limitations

A key strength of this study is its multiple case design, capturing 67 perspectives from patients, care partners, clinicians, and administrators across three U.S. clinics where recordings are used routinely. While generalizability is limited, findings align with those from controlled settings. We included a small number of interviews with individuals who chose not to record—this is an area for future research. Though the study was conducted some time ago, it remains timely and relevant. Despite technological advances, such as the emergence of ambient AI, the logistical aspects of making a clinic recording, listening, and sharing remain unchanged, and routine visit recording and sharing are still uncommon in practice. If anything, health systems are on the verge of broadly disseminating the practice of sharing recordings, making the adoption of our findings prescient.

### Implications

Driven by advances in AI, visit recording will continue to become more commonplace in healthcare. Building on the work of Ryan et al^6^^,^^7^, our research highlights that a visit interaction is more than a transaction of medical information—the visit interaction provides emotional support, promotes self-reflection, fosters greater family engagement, and helps patients prepare for future visits. Implementation efforts should include strategies to promote these benefits. Ensuring Internet access and technology comfort, especially in rural communities, appears to be an important factor to address in future implementations. Additionally, not all visits may require recording, especially when the complexity of the visit is low. Additionally, strategies are needed to address the legitimate concerns that initially deter clinicians from routine recording.

## Conclusion

In early adopter clinics, a range of stakeholders supported recording clinical encounters and outlined many perceived benefits of this practice. The few concerns raised primarily focused on confidentiality and security issues; some clinicians voiced concerns about the impact on clinic workflow or the intrusiveness. Overall, the case studies suggest that recording is a promising practice that can enhance recall, deepen understanding, extend care beyond the clinic, and engage care partners in a meaningful way.

## Supplementary Material

ooag033_Supplementary_Data

## Data Availability

Due to IRB requirements, access to de-identified data from this project will be considered on a case-by-case basis.
