# Supplementary material for: Providing routine digital recordings of clinic visits to patients: a multiple-case study of three settings in the U.S
Source: JAMIA Open. 2026 Apr 3;9(2):ooag033. doi: 10.1093/jamiaopen/ooag033 (PMC13049191; doi:10.1093/jamiaopen/ooag033)

**Supplementary 1**. PCP Recording System Screenshots.

List of prior visit recordings


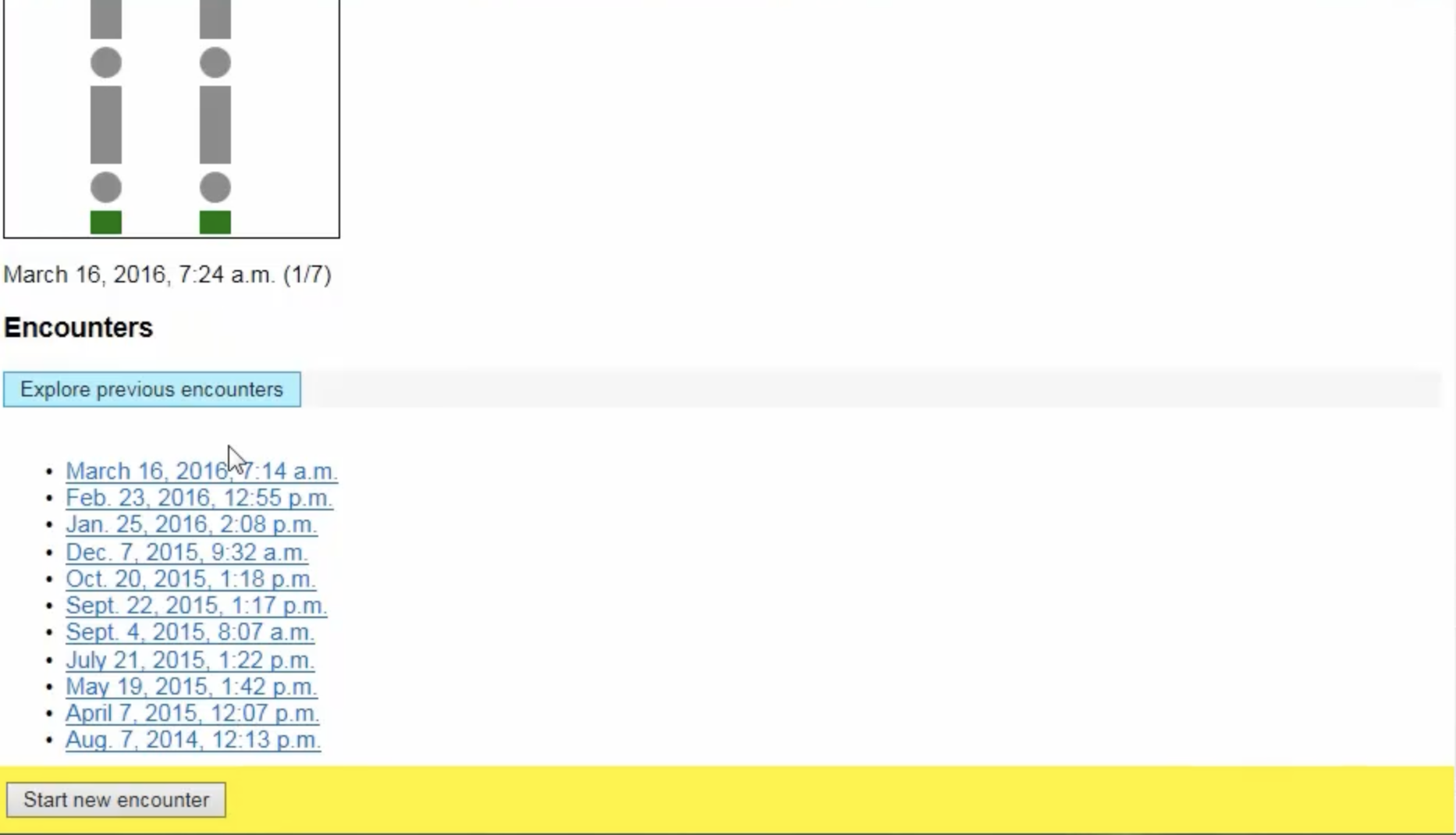


Recording play back


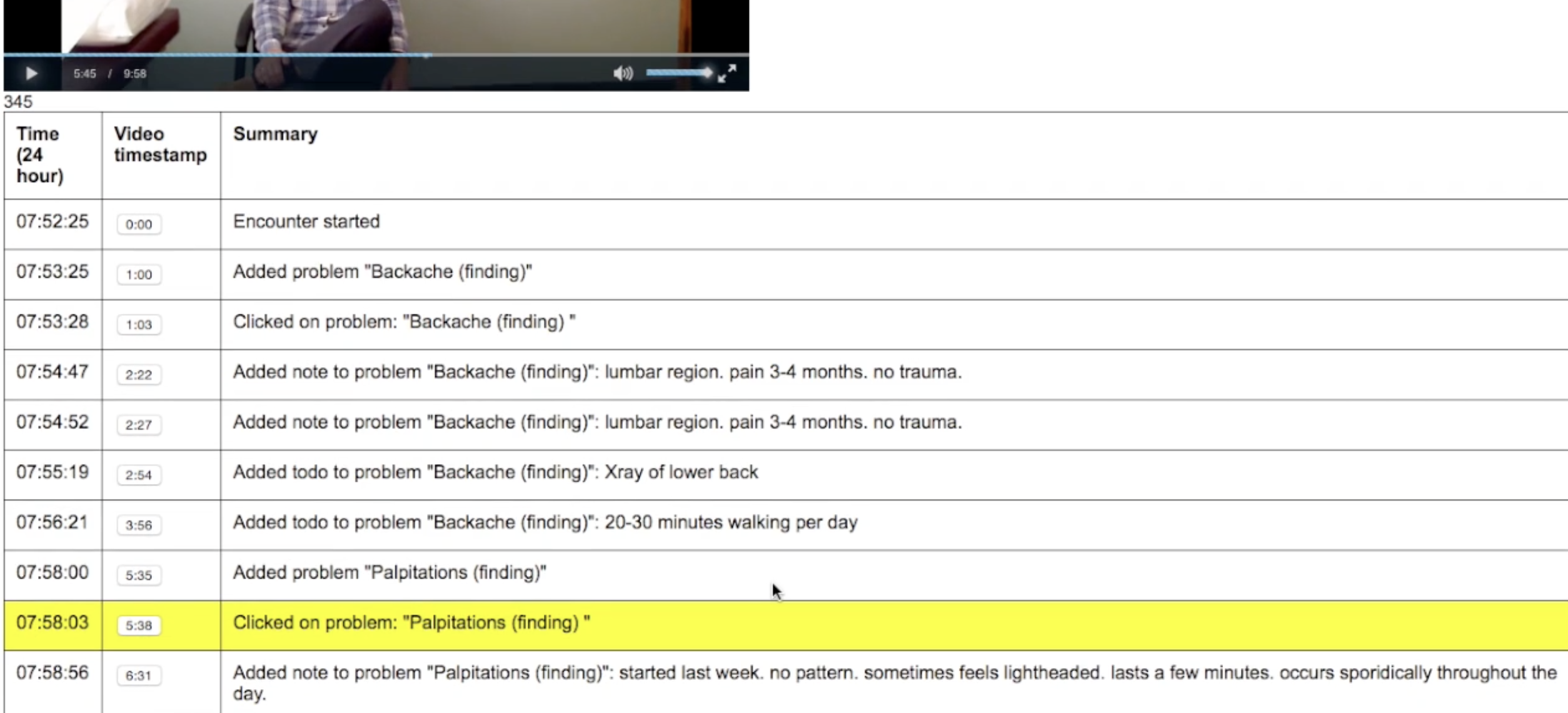


**Supplementary 2**. Cancer clinic recording advertising.
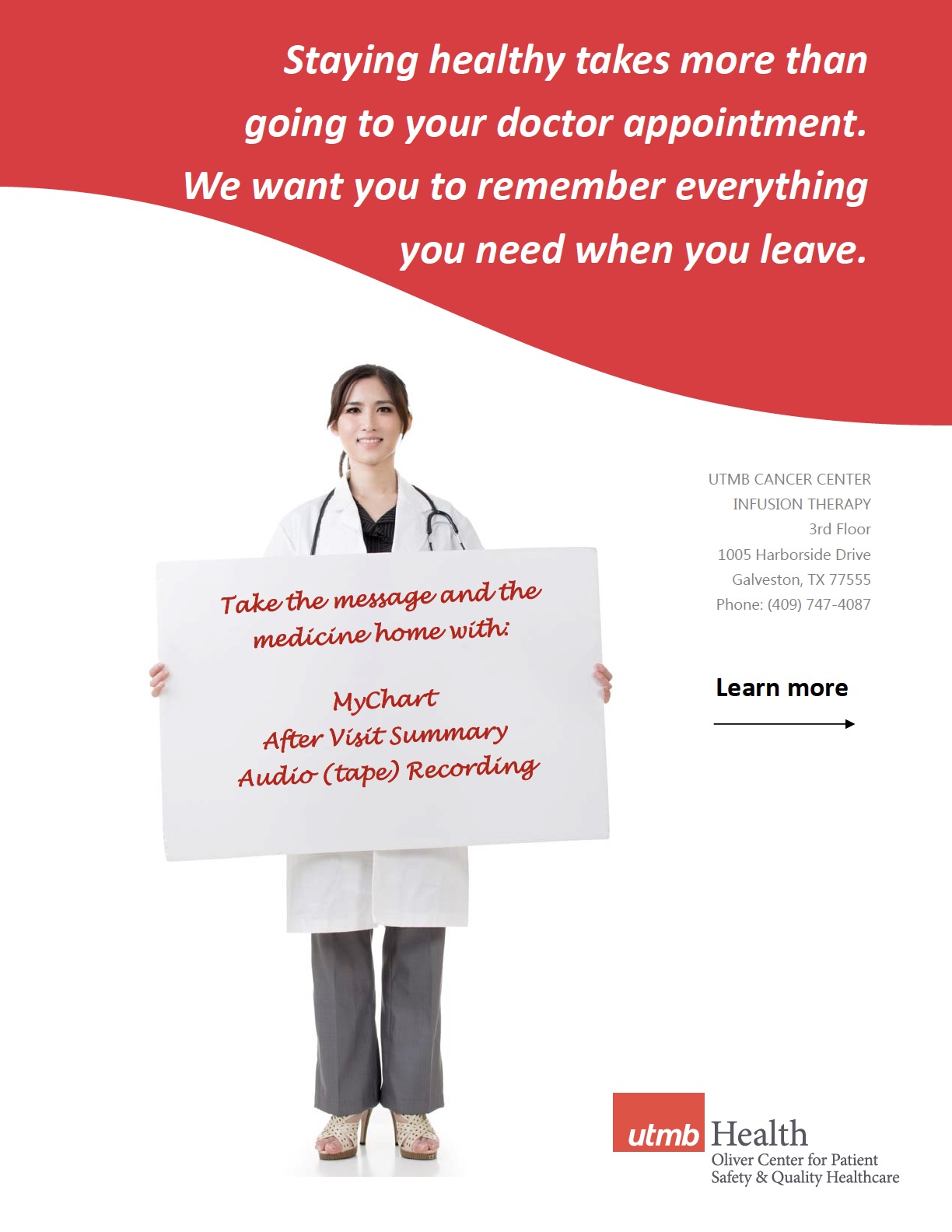

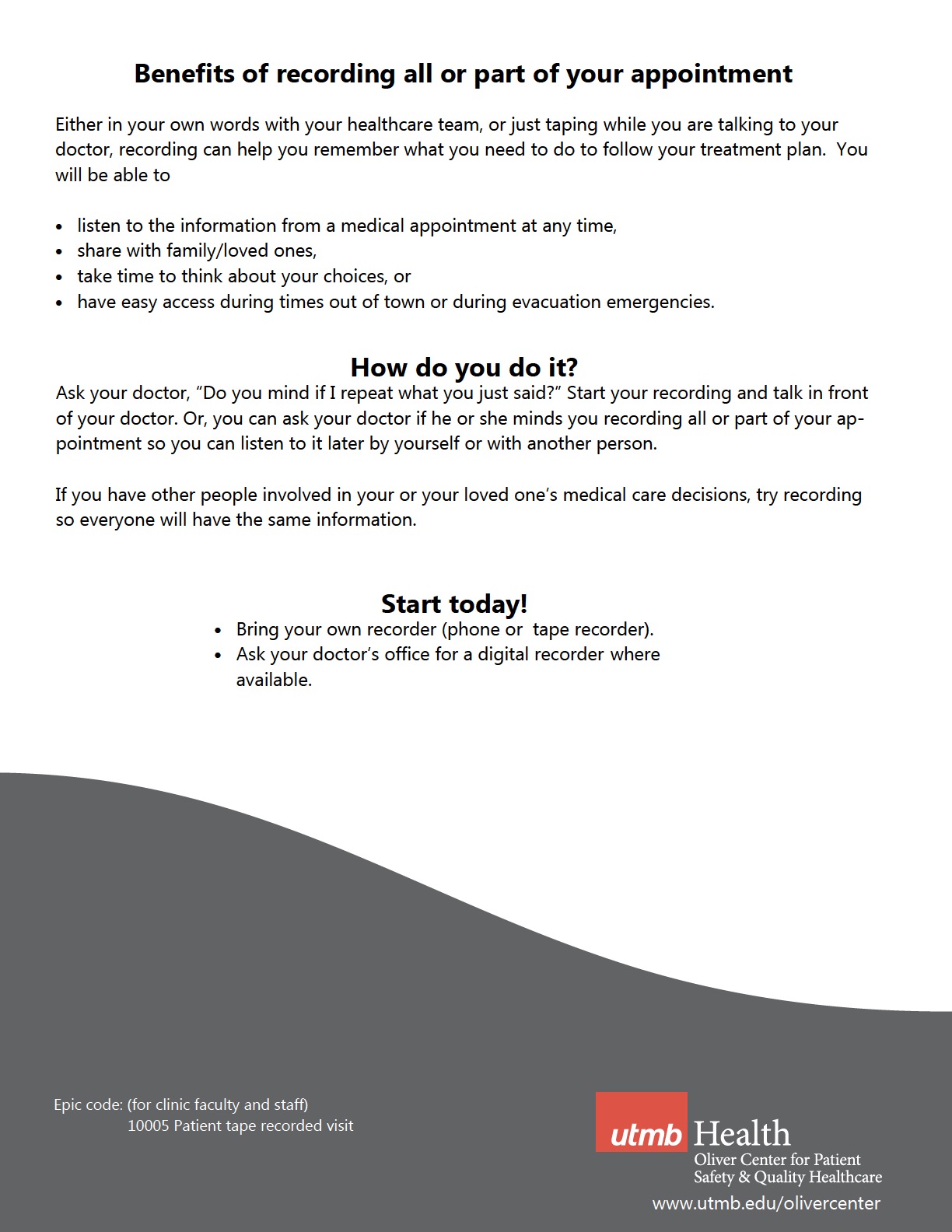


**Supplementary 3**. Neurology Recording System Screenshots.


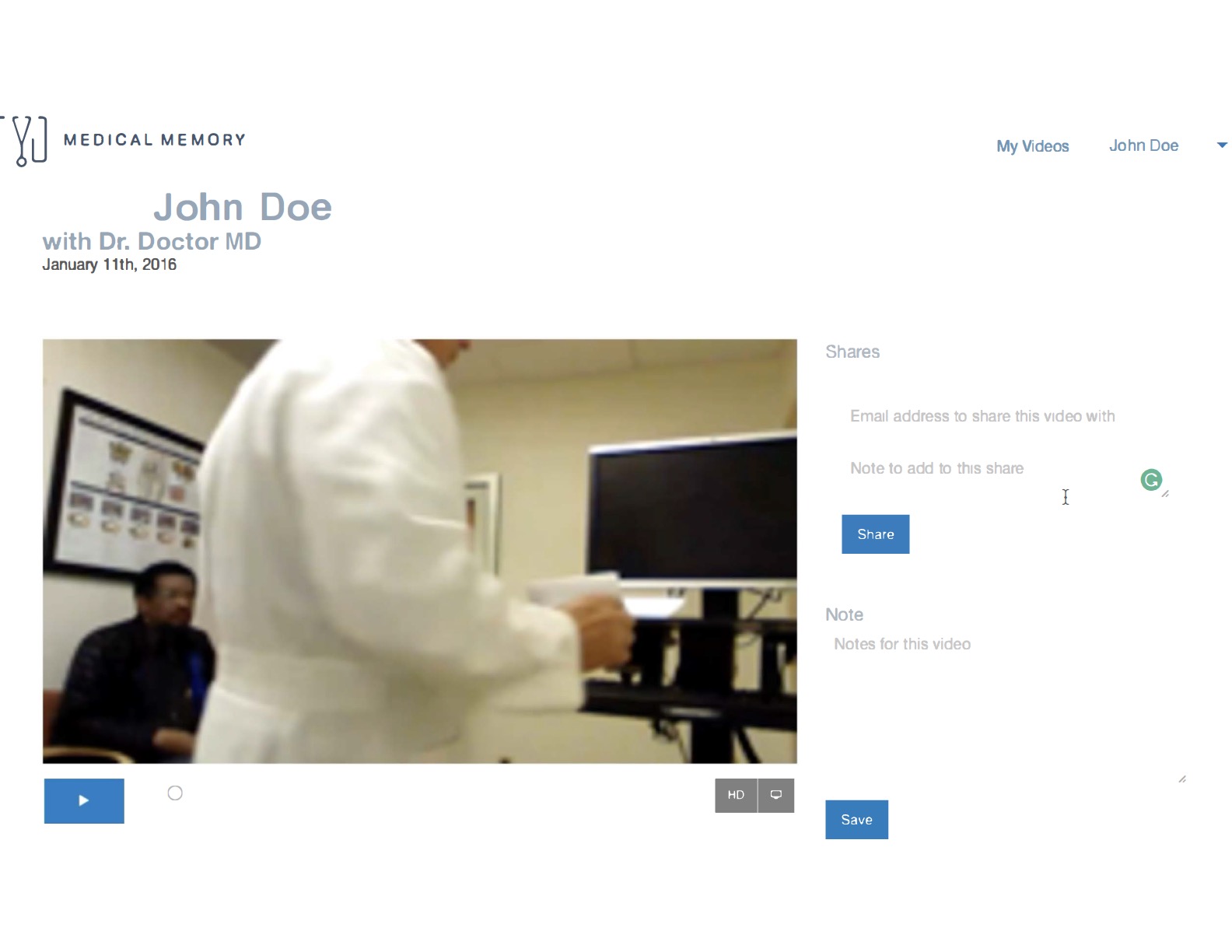


**Supplementary 4.** Neurology clinic recording help sheet.
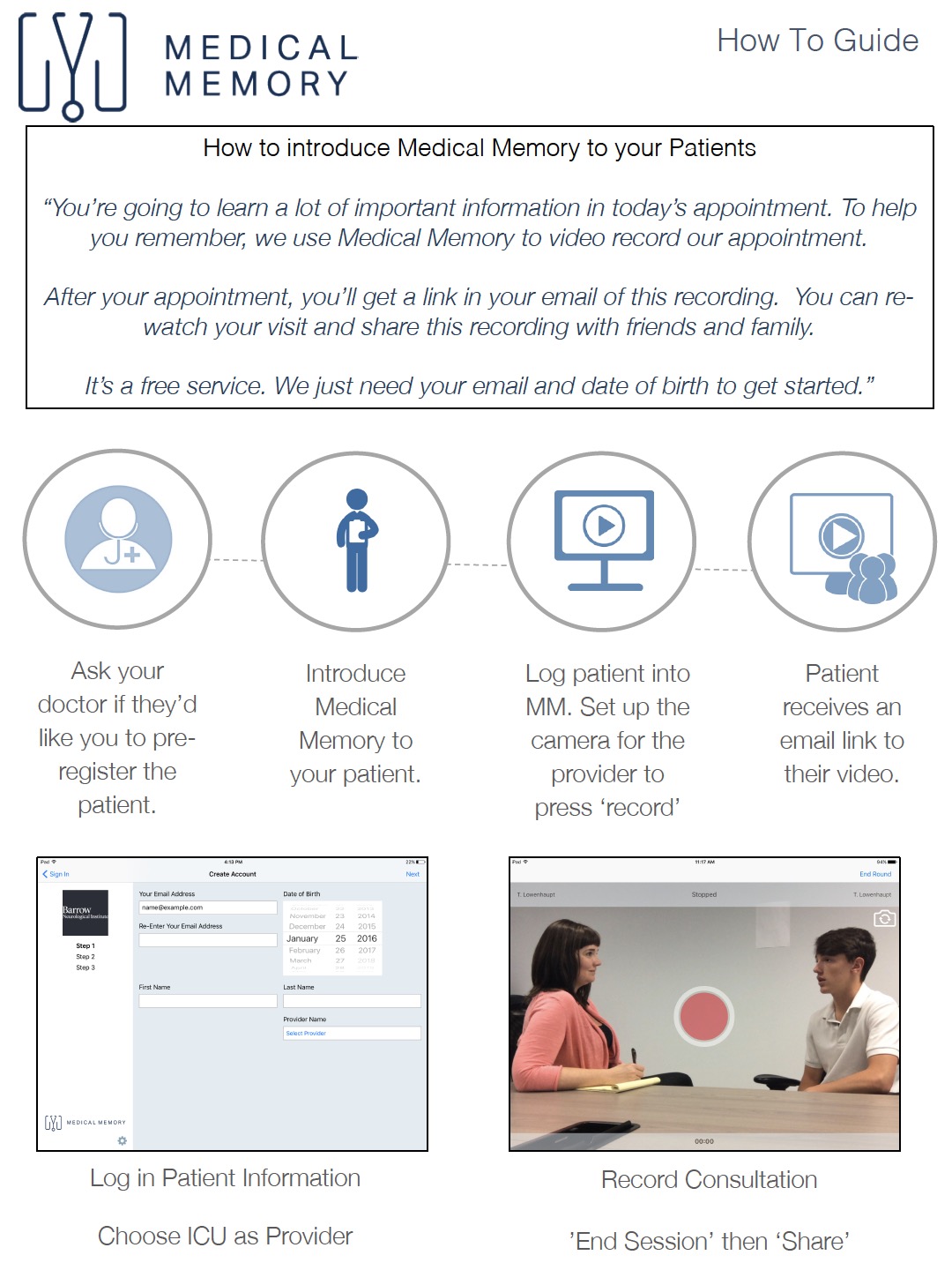

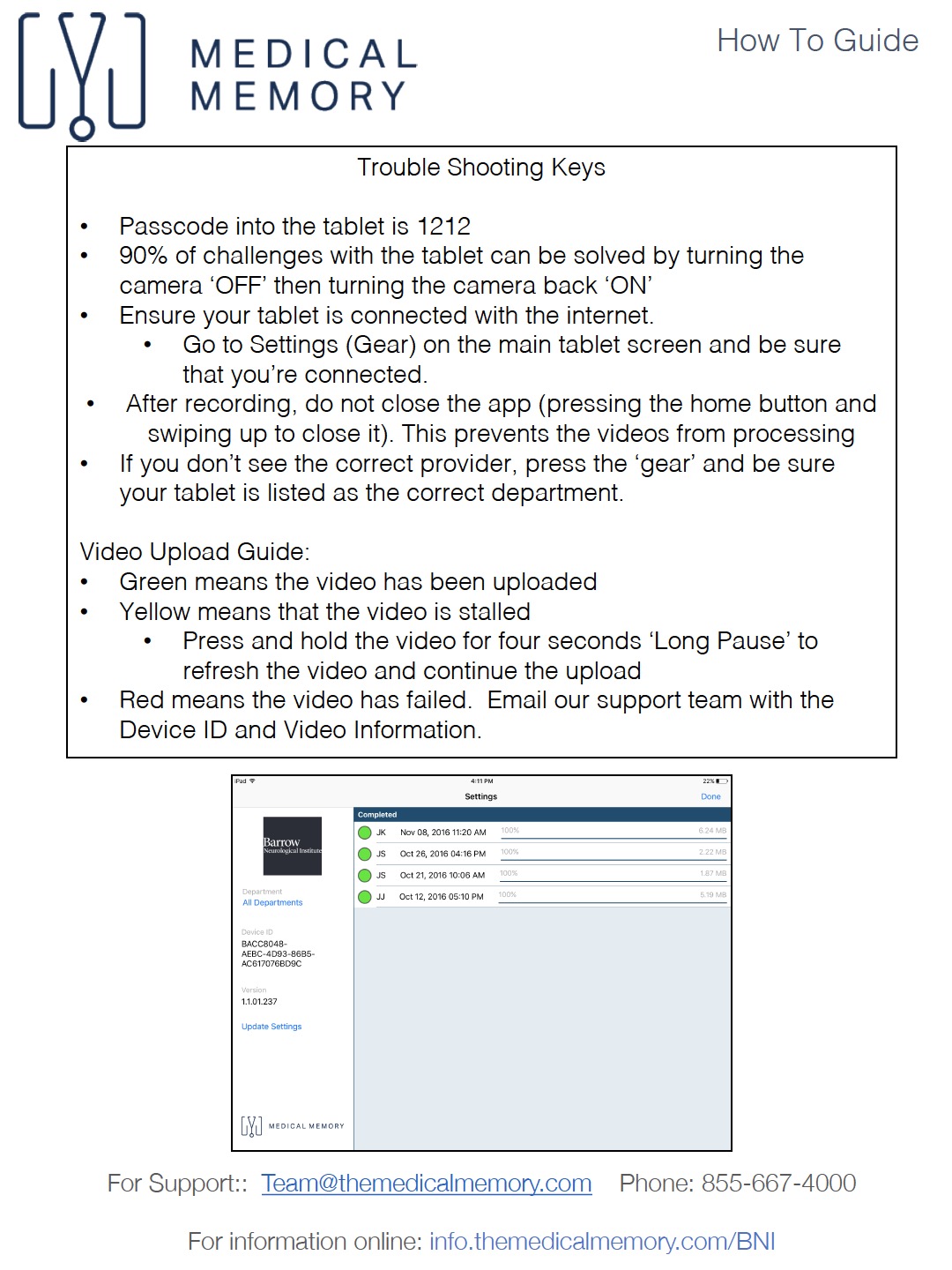

Supplement: ooag033_Supplementary_Data [file ooag033_supplementary_data.docx]
